# Arctic seabirds and shrinking sea ice: egg analyses reveal the importance of ice-derived resources

**DOI:** 10.1038/s41598-019-51788-4

**Published:** 2019-10-28

**Authors:** Fanny Cusset, Jérôme Fort, Mark Mallory, Birgit Braune, Philippe Massicotte, Guillaume Massé

**Affiliations:** 1 UMI Takuvik, Département de Biologie, Université Laval, 1045 Avenue de la Médecine, Québec, QC G1V 0A6 Canada; 2 LIENSs, UMR 7266, CNRS-La Rochelle Université, 2 Rue Olympe de Gouges, 17000 La Rochelle, France; 3 Biology Department, Acadia University, 15 University Avenue, Wolfville, NS B4P 2R6 Canada; 4 Environment and Climate Change Canada, National Wildlife Research Centre, Carleton University, Raven Road, Ottawa, ON K1A 0H3 Canada; 5 LOCEAN, UMR 7159, CNRS, MNHN, IRD, Sorbonne-Université, Station Marine de Concarneau, BP225, 29900 Concarneau, France

**Keywords:** Ecology, Climate-change ecology, Stable isotope analysis

## Abstract

In the Arctic, sea-ice plays a central role in the functioning of marine food webs and its rapid shrinking has large effects on the biota. It is thus crucial to assess the importance of sea-ice and ice-derived resources to Arctic marine species. Here, we used a multi-biomarker approach combining Highly Branched Isoprenoids (HBIs) with δ^13^C and δ^15^N to evaluate how much Arctic seabirds rely on sea-ice derived resources during the pre-laying period, and if changes in sea-ice extent and duration affect their investment in reproduction. Eggs of thick-billed murres (*Uria lomvia*) and northern fulmars (*Fulmarus glacialis*) were collected in the Canadian Arctic during four years of highly contrasting ice conditions, and analysed for HBIs, isotopic (carbon and nitrogen) and energetic composition. Murres heavily relied on ice-associated prey, and sea-ice was beneficial for this species which produced larger and more energy-dense eggs during icier years. In contrast, fulmars did not exhibit any clear association with sympagic communities and were not impacted by changes in sea ice. Murres, like other species more constrained in their response to sea-ice variations, therefore appear more sensitive to changes and may become the losers of future climate shifts in the Arctic, unlike more resilient species such as fulmars.

## Introduction

Sea ice plays a central role in polar marine ecosystems; it drives the phenology of primary producers that constitute the base of marine food webs. Primary producers provide the energy which is transferred to successive trophic levels, including zooplankton, fish, seabirds and marine mammals^1,2^. In the Arctic, primary production includes two consecutive pulses of marine autotrophs: sea-ice algae and phytoplankton^3^. In early spring, increasing irradiance and rising temperatures enable ice algae to grow. Later in the season when snow and sea ice melt, a phytoplankton bloom develops and follows the ice retreat^4,5^. Sea ice algae alone contribute 3–25% of the total primary production^6^, while in the central Arctic Ocean, this contribution could reach 57–83%^7,8^. In the last decades, the Arctic has changed rapidly as a result of anthropogenic climate change, facing particularly drastic changes in sea ice conditions^9^. One of the most visible changes in Arctic sea ice is a significant decrease in coverage^10,11^, with a mean rate of decline in summer extent of 12.8% per decade relative to the 1981–2010 average (National Snow and Ice Data Center), and similar trends are observed for the winter ice extent. Besides changes in coverage, Arctic sea ice has undergone a significant decrease in thickness^12^ with a shift from largely perennial to seasonal first-year ice cover^13^. Concurrently, earlier ice break-up^14,15^ and a prolonged melting season^16^ have produced changes in the open-water season. With these ongoing trends, climate models predict an ice-free Arctic Ocean in summer by 2050 or by the end of 21st century at the latest^9,17^. The productive period will thus start earlier and extend until later^18^, which will have strong implications on the phenology of primary producers^19^, ultimately impacting the total primary productivity of the Arctic^20^. For example, the primary production in the Arctic Ocean increased by 30% between 1998 and 2012^21^. Clearly, these changes will deeply modify the structure and the functioning of Arctic marine ecosystems^22^, in particular large community shifts and changes in range, abundance and growth of several species^18,23,24^. In such a context, it appears crucial to assess the dependency of key Arctic species on sea ice and its resources and to understand the risks associated with its current decline.

Biomarker approaches enable us to infer the dependency of Arctic consumers on both sympagic (i.e. sea ice-associated) and pelagic resources, by using conservative bioindicators produced by primary producers (ice-algae and phytoplankton respectively), then transferred along the food chain. Trophic biomarkers, such as stable isotopes and Highly Branched Isoprenoids (HBI), thus allow for tracking the trophic fingerprint of both primary producers. Stable isotopes of nitrogen (δ^15^N) and carbon (δ^13^C) provide information on the relative trophic level of consumers (i.e. diet) and the feeding habitat (i.e. benthic versus pelagic food chains), respectively^25^. In polar regions, the distinct δ^13^C signatures of ice algae (i.e. more enriched in ^13^C) and pelagic phytoplankton allow for reconstructing foraging on sympagic prey and can hence be considered as an ice proxy in the Arctic^26,27^. Additionally, HBI biomarkers provide complementary information on the relative contributions of the two primary production pools along with the food web^28,29^, namely sea ice algae and phytoplankton pools. HBIs are lipid biomarkers synthesized by diatom species almost exclusively belonging to genera *Haslea*, *Navicula*, *Rhizosolenia* and *Pleurosigma*^28^. In the Arctic, a restricted number of ice-associated diatom species produce IP_25_, a mono-unsaturated HBI isomer, which is often associated with a di-unsaturated isomer (diene)^30,31^. When observed in biological matrices, IP_25_ and diene provide direct evidence for the contribution of sympagic algae to the diet of the studied organisms. In contrast, a tri-unsaturated HBI (triene) is mostly synthesized by open-water diatom species, representing an indicator of the phytoplankton contribution. Both HBIs and stable isotopes, therefore, constitute powerful analytical tools for assessing environmental effects on marine biota and combining them would enable to better understand how much sea ice and its derived resources are important among Arctic marine ecosystems, from zooplankton consumers up to top predators, such as seabirds.

Seabirds represent a very large biomass^32^ and play a key role in the functioning of the Arctic marine ecosystems^33,34^. They represent excellent bioindicators of the marine environment^35,36^, as they are highly sensitive to changes in oceanographic conditions, food supply or pollution^37–39^, and thus could be largely impacted by changes in sea ice extent^40–43^. Further understanding about the role and importance of sea ice for these species is therefore required, especially since investigations involving HBIs and Arctic seabirds are currently missing.

Thick-billed murres (or Brünnich’s guillemot, *Uria lomvia*) and northern fulmars (*Fulmarus glacialis*) represent ideal candidates to investigate the importance of current and future Arctic sea ice conditions for seabirds. Both exhibit a near circumpolar distribution; they are amongst the most abundant seabird species in the Arctic during the breeding season and show contrasting feeding ecologies and flight constraints. Both are primarily “income breeders” relying on resources acquired just prior to breeding to form eggs^44,45^. While thick-billed murres are pursuit-divers feeding mainly on fish and macrozooplankton and present high flight costs^46^ restricting their large-scale movements, northern fulmars are opportunistic surface-feeders with limited flight constraints^42^. Given these contrasting characteristics, thick-billed murres and northern fulmars might be affected differently by changes in sea ice in areas surrounding their breeding grounds. Widely distributed in ice-free waters, northern fulmars are not tied to sea ice, whereas thick-billed murres largely breed in waters where sea ice occurs. We, therefore, hypothesize that sea ice would be more important to the breeding ecology of thick-billed murres. Previous studies highlighted negative impacts of a heavy ice cover on thick-billed murre breeding success^47,48^, resulting in delayed laying and smaller eggs^42,49^. Hence, the observed decline in Arctic sea ice extent and modifications in phenology could have strong implications on the fate of seabird populations. Surely, sea ice represents a physical barrier for seabirds excluding certain areas for foraging, and thus potentially increasing their energy expenditure to access prey in open water. However, an extensive ice cover does not impede them to access sympagic resources if several leads are present between ice floes that allow them to dive and feed. More importantly, sea ice could provide high-quality sympagic resources for breeding birds, and investigations on the profitability of sea ice are thus required.

In this study, we investigated the sensitivity of thick-billed murres (hereafter ‘murres’) and northern fulmars (hereafter ‘fulmars’) to variations in sea-ice conditions in the vicinity of their breeding grounds. More specifically we used a multi-biomarker approach combining HBIs and stable isotopes to: (i) determine if changes in sea ice conditions influence bird association with ice-derived resources; and (ii) investigate how this association influences bird feeding ecology and egg parameters (proxies of bird investment in the reproduction) of each species.

## Results

### Sea ice conditions around Prince Leopold Island (PLI) during the study period

**Figure 1:**
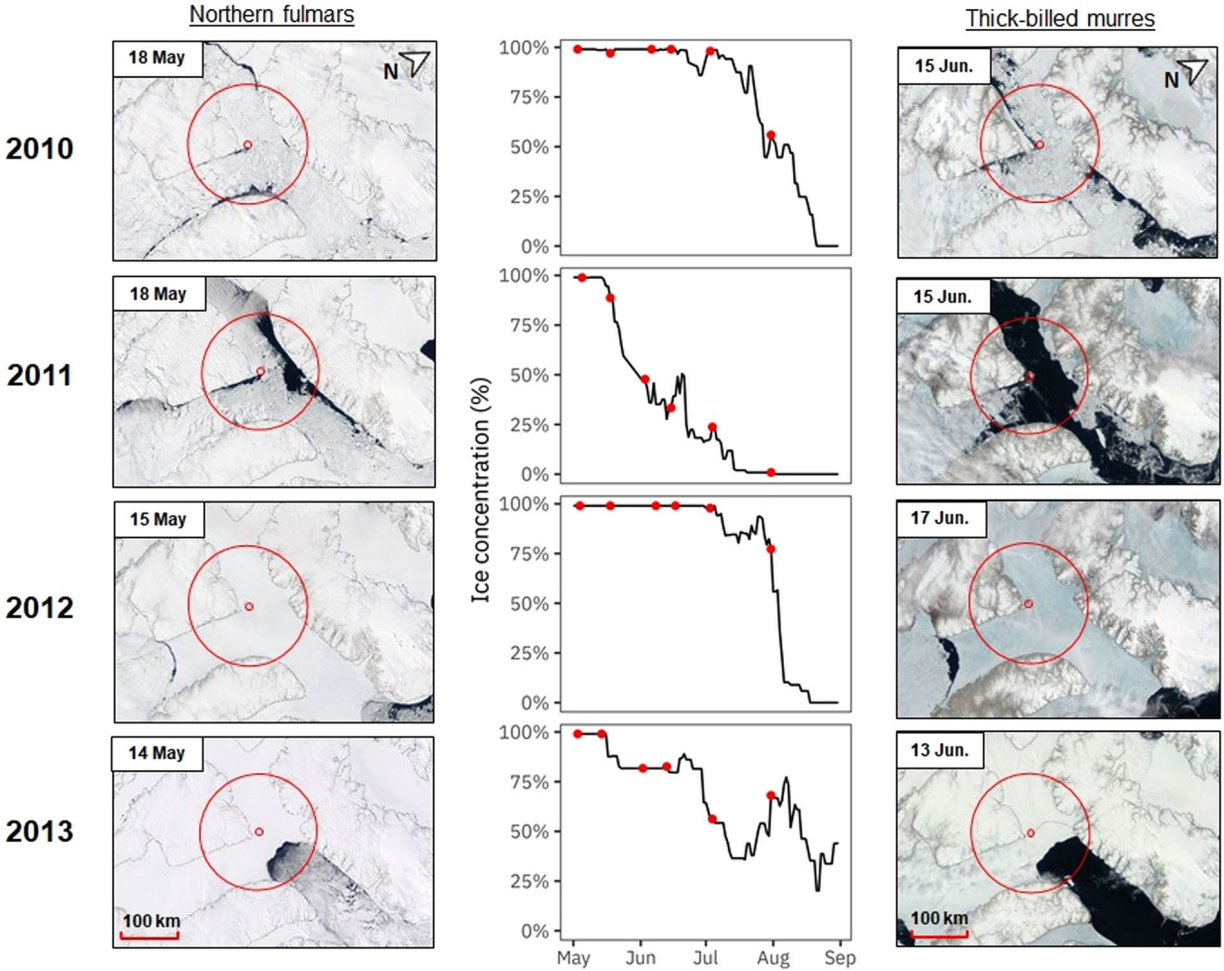
Ice conditions around Prince Leopold Island (inner red circle) in Barrow Strait (Nunavut, Canada) during egg formation of northern fulmars (left) and thick-billed murres (right) between 2010 and 2013. Satellites images (NASA Worldview, https://worldview.earthdata.nasa.gov/) highlight sea ice distribution in mid-May (for fulmars; https://worldview.earthdata.nasa.gov/?v=-1661127.7614493677,-1444657.9073818922,-856313.78481856,-1066353.4209707966&p=arctic&t=2010-05-18-T14%3A45%3A31Z) and in mid-June (for murres; https://worldview.earthdata.nasa.gov/?v=-1661127.7614493677,-1444657.9073818922,-856313.78481856,-1066353.4209707966&p=arctic&t=2010-06-15-T14%3A45%3A31Z). The outer red circle represents the 100 km radius circle around the colony, in which daily ice concentrations (center) were calculated for the entire breeding season (May to September). Red dots correspond to dates presented in Supplementary Fig. S1 for each reproductive event (i.e. colony attendance, egg-laying, egg-hatching).

Between 2010 and 2013, both northern fulmars and thick-billed murres encountered contrasting ice conditions around PLI during the entire breeding season (Supplementary Fig. S1), including egg formation (Fig. 1).

Once they attended the colony from early May, fulmar females form their single egg in mid-May, which is then laid in early-June. During mid-May, which thus corresponds to the egg formation period, ice concentrations ranged from 80% to 99% between 2010 and 2013 (Table 1a). In 2010, surrounding waters were covered by vast ice floes (2–10 km) of thick, first-year ice separated by numerous leads (i.e. access to water) (Fig. 1). In 2011, open-water was accessible between multiple big ice floes (500–2000 m) of thick first-year ice (>120 cm), but sea ice was relatively present. In 2012, consolidated fast ice combining thick first-year ice (>120 cm) and new ice (<10 cm) surrounded the island. The ice margin was located far away (>250 km to the east). In 2013, a dense and thick first-year fast ice (>120 cm) was present around PLI but open water remained accessible near the colony (~50 km).

**Table 1:** Ice conditions in a 100 km radius circle from Prince Leopold Island (Nunavut, Canada) during the breeding season of northern fulmars (starting in May) and thick-billed murres (starting in June) between 2010 and 2013.

Year	2010	2011	2012	2013	Reproduction
***(a) Northern fulmars***					
Ice concentrations					
Early-May (%)	92.4	98.4	99.0	97.0	Colony attendance
**Mid-May (%)**	**95**.**6**	**80**.**2**	**99**.**0**	**93**.**3**	Departure for exodus
Early-June (%)	97.6	42.8	98.9	83.1	Egg laying
End-July (%)	39.1	0.80	12.3	40.8	Egg hatching
Ice description (Early-May)	Patchy, large floes, access to open-water	Patchy, large floes, access to open-water	Dense pack ice, ice edge far away (>250 km E)	Dense pack ice, but ice margin close (50 km)	
***(b) Thick-billed murres***					
Ice concentrations					
**Mid-June (%)**	**92**.**6**	**29**.**1**	**98**.**8**	**79**.**0**	Colony attendance
Early-July (%)	79.4	11.2	88.9	46.2	Egg laying
End-July (%)	39.1	0.80	12.3	40.8	Egg hatching
Ice description (Mid-June)	Patchy, large floes, access to open-water	Open-water, ice-free	Dense pack ice, ice edge far away (>250 km E)	Dense pack ice, but ice margin close (50 km)	

Once they attended the colony, fulmars leave it temporarily between mid- and end-May for their ≪pre-laying exodus≫, before returning for egg-laying. Egg formation is the critical phase investigated in this study and occurs in mid-May and mid-June for fulmars and murres, respectively (in bold). Despite the temporal lag in their reproductive phenology, chicks of both species hatch around end-July.

Murres only arrive at the colony between early- and mid-June and lay their single egg at the end of June or start of July (i.e. approximately one month later than fulmars). In mid-June, which corresponds to the egg formation period, sea ice concentrations ranged from 29% to 99% during the study period (Table 1b). In 2010, patches of sea ice were present around the colony, but patches of open water were easily accessible. Vast ice floes (2–10 km) of thick first-year ice (>120 cm) were surrounded by large leads (Fig. 1). In contrast, open-water conditions were present in the entire region in 2011. Fast ice was only present in coastal areas close to Devon Island shores, as medium-sized (100–500 m) but sometimes large ice floes (2–10 km) of thick first-year ice (>120 cm). In 2012, the colony was still surrounded by a dense and consolidated fast ice (medium and thick first-year ice, 70− 120+ cm) and the ice edge was still located more than 250 km away to the east (Lancaster Sound Polynya). Finally, in 2013, although the area was surrounded by dense fast ice with thick first-year ice (>120 cm), the ice edge was easily accessible located 50 km away from PLI.

Thus, we distinguished two major ice regimes (Fig. 1): (1) 2010 and 2012 represented heavy ice years, with a late ice break-up (i.e. in July) and an extensive ice cover, and (2) 2011 and 2013 were defined by an early ice break up (i.e. in May) and a restricted sea ice cover or an easy access to open water. However, we noticed a gradient over the years from icy to open-water conditions, gradually from 2012 to 2010, 2013 and 2011 for murres (mid-June) and from 2012 to 2010, 2013 and 2011 for fulmars (mid-May).

### Biological markers as proxies for sea ice association and use of ice-derived resources

#### Ice use index

**Figure 2:**
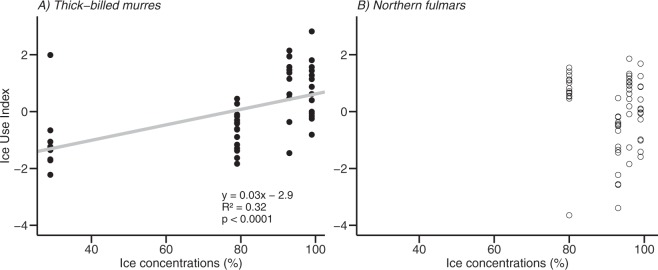
Relationship between ice use index and ice concentrations in the 100 km radius circle around the colony for thick-billed murres (TBMU, black circles) and northern fulmars (NOFU, open circles) between 2010 and 2013.

In murres, the ice use index differed significantly across years (*F*_3,40_ = 12.1, *p* < 0.001, ANOVA), with 2010 (1.0 ± 1.1 SD) and 2012 (0.7 ± 1.0) differing from 2011 (−1.1 ± 1.1) and 2013 (−0.7 ± 0.7), and increased linearly with increasing ice concentrations (p < 0.001, *R*² = 0.32; Fig. 2). In fulmars, the ice use index was significantly different across years (*F*_3,52_ = 8.8, *p* < 0.001, ANOVA), with 2013 (−1.1 ± 1.1) differing from 2010 (0.6 ± 1.0), 2011 (0.6 ± 1.2) and 2012 (−0.0 ± 1.0), and did not vary linearly with ice concentrations (*p* > 0.05).

#### Highly branched isoprenoids

The three HBI isomers (i.e. IP_25_, diene, triene) were present in all samples, but their concentrations were highly variable between individuals (Supplementary Fig. S2). HBI concentrations were very low in fulmar eggs (IP_25_ = 2.5 ± 2.7 ng.g^−1^ sample, diene = 0.4 ± 2.7 ng.g^−1^ standard, triene = 0.4 ± 0.6 ng.g^−1^ standard) compared to those observed in murre eggs (IP_25_ = 7.6 ± 11.2 ng.g^−1^ sample, diene = 3.4 ± 6.7 ng.g^−1^ standard, triene = 4.1 ± 4.5 ng.g^−1^ standard) (Supplementary Fig. S3).

**Figure 3:**
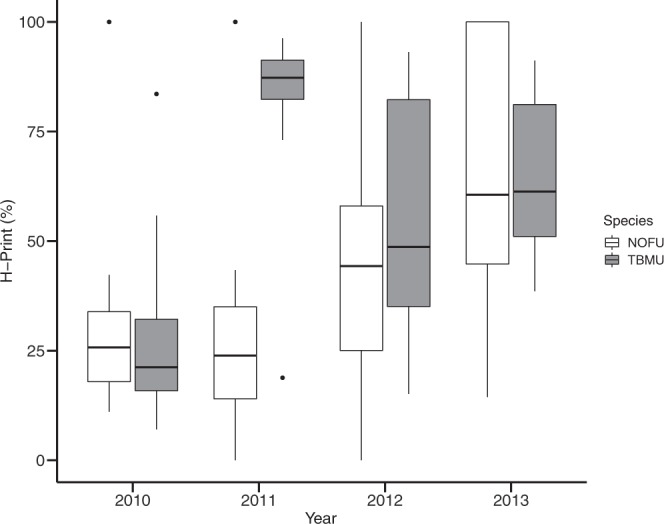
H-Print (%) for eggs of thick-billed murres (grey) and northern fulmars (white) (n = 15/year/species) collected on Prince Leopold Island for four consecutive years with contrasted ice conditions (2010–2013).

For murres, HBI concentrations varied among years, ranging overall from 0 to 38.2 (ng.g^−1^ standard) and H-Print differed significantly across years (*F*_3,40_ = 8.9, *p* < 0.001, ANOVA; Fig. 3), with 2010 standing out from all other years with significantly lower values. In 2010, ice specific isomers were abundant (IP_25_ = 18.9 ± 17.4 ng.g^−1^ sample, diene = 9.7 ± 11.3 ng.g^−1^ standards; Supplementary Fig. S2a), with the lowest H-Print of all years (28.1 ± 20.8%, Table 2). In 2011, the H-Print was the highest (82.5 ± 18.8%) reflecting the high triene abundance (6.4 ± 6.5 ng.g^−1^ standard). In 2012, ice association differed between two groups (*p* < 0.001, *t*_11 9_ = −8,4, Welch two-sample *t*-test): one group with higher amounts of ice biomarkers (H-Print = 34.1 ± 10.9%, n = 8) and a second group with higher amounts of pelagic biomarker (H-Print = 83.0 ± 10.2%, n = 7). Finally, in 2013 eggs exhibited an intermediate H-Print (63.4 ± 18.1%).

**Table 2:** Mean ± SD for the different trophic tracers, sea ice biomarkers and reproductive investment indicators measured for thick-billed murres and northern fulmars between 2010 and 2013 on Prince Leopold Island.

Variables	Thick-billed murres				Northern fulmars			
	2010	2011	2012	2013	2010	2011	2012	2013
**HBIs**								
IP_25_ (ng/g sample)	18.9 ± 17.4	1.8 ± 2.2	5.3 ± 3.7	4.5 ± 4.3	3.3 ± 3.1	3.4 ± 2.4	2.0 ± 2.0	1.4 ± 2.7
Diene (ng/g standard)	9.7 ± 11.3	0.9 ± 1.1	1.9 ± 2.0	1.1 ± 0.7	0.5 ± 0.5	0.6 ± 0.5	0.2 ± 0.2	0.3 ± 0.6
Triene (ng/g standard)	2.8 ± 2.0	6.4 ± 6.5	4.2 ± 4.7	2.9 ± 2.5	0.3 ± 0.2	0.3 ± 0.1	0.2 ± 0.2	0.7 ± 1.2
H-Print (%)	28.1 ± 20.8	82.5 ± 18.8	56.9 ± 27.3	63.4 ± 18.1	33.9 ± 28.1	27.3 ± 23.9	47.6 ± 33.1	67.6 ± 29.7
**Stable isotopes**								
δ^13^C (‰)	−19.7 ± 0,4	−20.3 ± 0.5	−19.3 ± 0.4	−20.3 ± 0.3	−19.3 ± 0.2	−19.4 ± 0.3	−19.4 ± 0.2	−19.7 ± 0.2
δ^15^N (‰)	16.0 ± 0.5	15.3 ± 0.7	15.8 ± 0.5	15.4 ± 0.8	13.0 ± 0.3	13.0 ± 0.3	13.4 ± 0.3	13.2 ± 0.3
**Ice Use Index**	1.0 ± 1.1	−1.1 ± 1.1	0.7 ± 1.0	0.7 ± 0.7	0.6 ± 1.0	0.6 ± 1.2	−0.0 ± 1.0	−1.1 ± 1.1
**Egg parameters**								
Egg volume (cm³)	191.9 ± 12.8	186.4 ± 16.1	199.4 ± 18.4	183.2 ± 17.6	177.4 ± 13.8	178.6 ± 14.6	173.4 ± 17.0	178.4 ± 11.7
Egg energy content (kcal)	127.5 ± 21.8	107.2 ± 38.4	156.1 ± 21.2	107.2 ± 22.4	—	—	—	—

For fulmars, the H-Print exhibited a significant inter-annual variability (*F*_3,52_ = 6.6, *p* < 0.001, ANOVA; Fig. 3), with 2013 differing from 2010 and 2011, where ice biomarkers were more abundant (i.e. lower H-Print, 33.9 ± 28.1% in 2010 and 27.3 ± 23.9% in 2011). In 2012 samples, H-Print values were intermediate (47.6 ± 33.1%) while in 2013 triene was more dominant in the eggs (i.e. higher H-Print, 67.6 ± 29.7%).

There was a significant positive linear relationship between H-Print and ice concentrations around the colony (*p* < 0.001, *R*² = 0.28) in murres, in contrast to fulmars for which no relationship was found (*p* > 0.05).

#### Stable isotopes

Stable isotope results confirmed the distinct feeding ecology of each species with murres exhibiting higher δ^15^N values (15.6 ± 0.67‰) than fulmars (13.2 ± 0.33‰) (*p* < 0.001, *t*_72.3_ = −23.9, Welch two-sample *t*-test; Supplementary Fig. S4).

**Figure 4:**
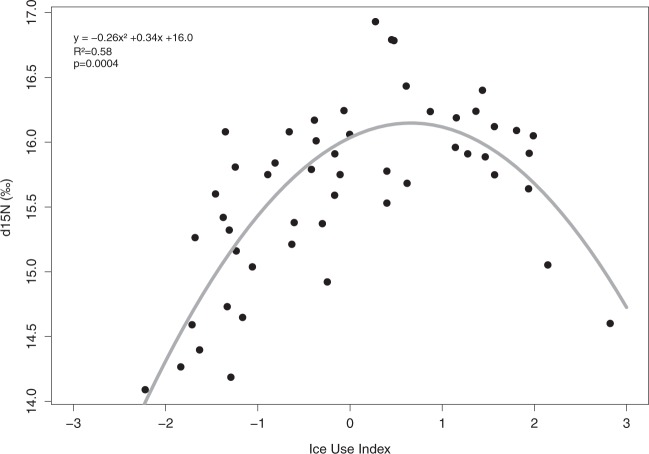
Influence of ice association (ice use index) on δ^15^N ratios measured in eggs of thick-billed murres between 2010 and 2013.

For both species, we identified distinct isotopic niches (MANOVA; murres: *F*_3,40_ = 5.8, *p* < 0.001; fulmars: *F*_3,56_ = 7.2, *p* < 0.001). For murres, δ^13^C values were significantly different between years (Table 2; *F*_3,40_ = 18.5, *p* < 0.001, ANOVA), with 2010 and 2012 differing from 2011 and 2013. δ^15^N were also different between years (*F*_3,40_ = 4.0, *p* = 0.015, ANOVA), with 2012 differing from 2011. In icy years, murre eggs were enriched in both ^15^N (16 ± 0.5‰ in 2010, 15.8 ± 0.5‰ in 2012) and ^13^C (−19.7 ± 0.4‰ in 2010, −19.3 ± 0.4‰ in 2012) compared to years characterized by open-water presence (δ^15^N = 15.3 ± 0.7‰, δ^13^C = −20.3 ± 0.5‰ in 2011; δ^15^N = 15.4 ± 0.8‰, δ^13^C = −20.3 ± 0.3‰ in 2013). For this species, δ^15^N values reached a maximum when the ice use index was intermediate (quadratic regression, *p* < 0.001, *R*² = 0.58; Fig. 4).

For fulmars, δ^13^C values were different across years (Table 2; *F*_3,52_ = 6.8, *p* < 0.001, ANOVA), with 2013 differing from all other years (−19.7 ± 0.2‰ versus −19.3 ± 0.2‰ in 2010, −19.4 ± 0.3‰ in 2011 and −19.4 ± 0.2‰ in 2012). δ^15^N values also differed across years (*F*_3,52_ = 7.0, *p* < 0.001, ANOVA), with 2012 (13.4 ± 0.3‰) different from 2010 (13.0 ± 0.3‰) and 2011 (13.0 ± 0.30‰). For this species, however, δ^15^N did not linearly respond to varying ice use index (*p* = 0.9).

### Sea ice and seabird egg parameters

**Figure 5:**
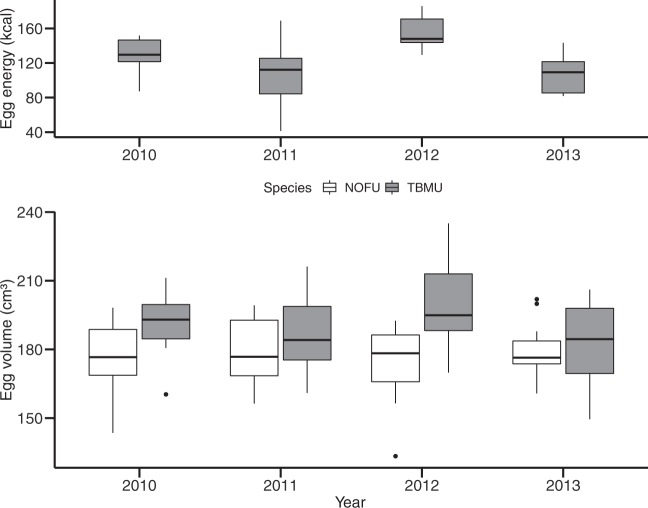
Egg volume (cm³) and egg energetic content (kcal) of northern fulmars (NOFU, white) and thick-billed-murres (TBMU, grey) between 2010 and 2013.

**Figure 6:**
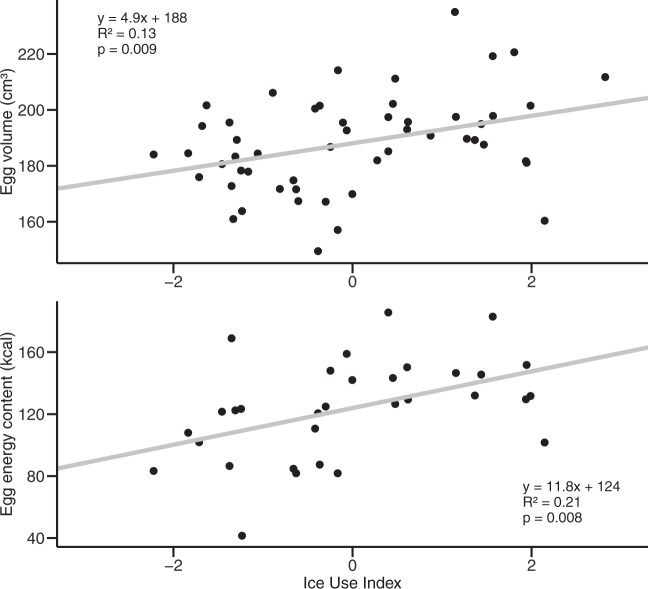
Influence of ice association (ice use index) on egg parameters of thick-billed murres: egg volume (top) and egg energetic content (bottom).

To investigate how variations in sea ice conditions and sea ice use influenced bird investment in the reproduction and egg quality, egg volumes (for both species) and egg energy contents (only for murres) were both compared to the ice use index. First, the egg volume did not differ significantly across years for murres (190.0 ± 17.0 cm^3^; *F*_3,56_ = 2.5, *p* = 0.07, ANOVA; Fig. 5). However, when analyzed by ice regime, eggs were on average larger in icy years (191.9 ± 12.8 cm^3^ in 2010 and 198.4 ± 18.7 cm^3^ in 2012) than in years of open-water (186.4 ± 16.1 cm^3^ in 2011 and 183.2 ± 17.6 cm^3^ in 2013) (*p* = 0.02, *t*_57_._9_ = 2,5, Welch two-sample *t*-test). Egg volume of murres increased linearly with increasing ice use index (*p* < 0.01, *R*² = 0.13; Fig. 6). For 2012, unlike for the H-Print, murres did not form distinct groups for their egg volume (*p* = 0.08, *t*_11 8_ = 1.9). For fulmars, egg volume remained stable across years (177.1 ± 14.0 cm³; *F*_3,44_ = 0.7, *p* = 0.54, ANOVA; Fig. 5) and did not linearly respond to changing ice use (*p* = 0.71; Table 3).

**Table 3:** Summary of statistical results in the present study for both thick-billed murres and northern fulmars.

Test	Variables	Parameters	
		Thick-billed murres	Northern fulmars
**MANOVA**	δ^13^C, δ^15^N	*p* < 0.001***	*p* < 0.001***
**ANOVA:** ~ Year	H-Print	*p* = 0.001***, *F*_3,40_ = 8.9, *n* = 11	*p* < 0.001***, *F*_3,52_ = 6.6, *n* = 14
	δ^13^C	*p* < 0.001***, *F*_3,40_ = 18.5, *n* = 11	*p* < 0.001***, *F*_3,52_ = 6.7, *n* = 14
	δ^15^N	*p* = 0.01*, *F*_3,40_ = 3.9, *n* = 11	*p* < 0.001***, *F*_3,52_ = 7.0, *n* = 14
	Ice Use Index	*p* < 0.001***, *F*_3,40_ = 12.1, *n* = 11	*p* < 0.001***, *F*_3,52_ = 8.8, *n* = 14
	Volume	*p* < 0.07, *F*_3,56_ = 2.5, *n* = 15	*p* = 0.5, *F*_3,44_ = 0.7, *n* = 12
	Energy content	*p* = 0.01*, *F*_3_,_24_ = 4.6, *n* = 7	—
**Welch two-sample t-test**			
~ Species	δ^15^N	*p* < 0.001***, *t* = −23.9; Mean (TBMU) = 15.6	Mean (NOFU) = 13.2
~2012 Groups	H-Print	*p* < 0.001***, *t* = −8.4; Mean (Ice) = 35.4; Mean (Open-water) = 83.0	—
	Volume	*p* = 0.08, *t* = 1.9; Mean (Ice) = 203.2; Mean (Open-water) = 188.3	—
	Energy content	*p* = 0.8, *t* = −0.3; Mean (Ice) = 152.6; Mean (Open-water) = 158.6	—
~Year groups	Volume	*p* = 0.01*, *t* = 2.5; Mean (Cold) = 195.2; Mean (Warm) = 184.8	—
	Energy content	*p* = 0.002**, *t* = 3.3; Mean (Cold) = 140.0; Mean (Warm) = 107.2	—
**Linear regression**			
~ Ice concentrations	H-Print	*p* < 0.001***; y = −0.55x + 99.0; *R*² = 28.2	*p* > 0.05 (Non-significant)
	Ice Use Index	*p* < 0.001***; y = 0.03x− 2.1; *R*² = 32.1	*p* > 0.05 (Non-significant)
~Ice Use Index	δ^15^N	*p* < 0.001***; y = 0.25x + 15.6; *R*² = 22.4	*p* = 0.9 (Non-significant)
	Egg volume	*p* = 0.009**; y = 4.9x + 188.1; *R*² = 13.2	*p* = 0.7 (Non-significant)
	Egg energy content	*p* = 0.008**; y = 11.8x + 123.9; *R*² = 21.2	—
~δ^15^N	Egg volume	*p* = 0.1 (Non-significant)	*p* = 0.09 (Non-significant)
	Egg energy content	*p* = 0.005**; y = 24.0x − 253.3; *R*² = 23.4	—
~Egg volume	Egg energy content	*p* = 0.024*; y = 0.81x − 25.8; *R*² = 15.9	—
**Quadratic relationship**	δ^15^N ~ Ice Use Index	*p* < 0.001***; y = −0.26 x² + 0.34x + 16.0; *R*² = 58.1	—

The energy content of murre eggs differed significantly across years (*F*_3,24_ = 4.6, *p* = 0.01, ANOVA), with higher energetic content in icier years (127.5 ± 21.8 kcal in 2010 and 156.1 ± 21.2 kcal in 2012) than in open-water years (107.2 ± 38.4 kcal in 2011 and 107.2 ± 22.4 kcal in 2013) (*p* = 0.02, *t*_29 1_ = 3.3; Fig. 5), and with 2012 differing from 2011 and 2013. As for the egg volume, the two distinct groups of murres in 2012 did not show distinct egg energetic content (*p* = 0.8, *t*_3.4_ = −0.3). The energetic content linearly increased as the ice use index increased (*p* < 0.01, *R*^2^ = 0.21; Fig. 6). There was also a linear positive relationship between energetic content and δ^15^N values (*p* = 0.005, *R*^2^ = 0.23; Supplementary Fig. S5). Finally, the energetic content linearly increased with increasing egg volume (*p* = 0.02, *R*^2^ = 0.16; Supplementary Fig. S6).

## Discussion

Sea ice phenology, by shaping the pulses of primary productivity in the Arctic, may control the ecology and reproductive success of seabird species^50^. In this study, variations in the abundance and distribution of HBIs^51^ and stable isotopes in eggs were used to determine the influence of sea ice on diet and breeding investment of two key Arctic seabirds. We confirmed that HBIs acquired *via* food are transferred into eggs during their production by female birds. This transfer was previously reported for two iconic Antarctic bird species, Adelie penguins (*Pygoscelis adeliae*) and snow petrels (*Pagodroma nivea*)^52^. Our results, therefore, provide additional insights into HBI transfer to the highest trophic levels across the marine food web in the Arctic, even if the metabolism, the assimilation and the elimination processes still remain poorly understood. The presence of IP_25_ and diene in egg samples reflects the contribution of ice-derived organic matter in the diet of females while the contribution of the organic matter produced in open waters is represented by the triene. With ice- and plankton-derived isomers observed in all samples (Supplementary Fig. S2), our investigations demonstrate that both species rely on both primary production pools. However, the large interspecific and inter-annual differences (Supplementary Fig. S3) observed reveal that thick-billed murres and northern fulmars differed significantly in their relationship to sea ice and its associated resources.

### Bird relationship to sea ice

During icy years, murres laid eggs with biomarker (HBI and carbon stable isotopes) patterns suggesting high ice use, in contrast to biomarker patterns from low ice years. The positive linear relationships observed between ice concentrations and ice use index suggest that murres strongly respond to changes in sea ice and would thus be more sensitive to potential impacts of climate change.

Amongst the four years studied, 2012 was the iciest year. The entire region around Prince Leopold Island (PLI) was covered by a dense, thick and unfragmented fast ice that remained until very late in the season. With the highest ice use indices during 2012 on average, birds were relying the most on sea ice for feeding (Fig. 2). High ice use indices are also observed in 2010 samples and sea ice was also very abundant in the area that year. This further confirms the strong relationship between murres and sea ice around PLI. Interestingly, HBIs were up to six times more abundant in eggs collected in 2010 than in those from 2012 (Supplementary Fig. S2). In fact, in 2012 the ice margin was located at the entrance of Lancaster Sound until mid-July. Access to open water was thus limited and birds had to travel longer distances to access their feeding grounds (conditions previously shown to be deleterious to breeding at this colony^42^). Also abundant in 2010, sea ice was however fragmented in relatively large and mobile floes separated by numerous leads. This configuration provided easy access to water and its resources for murres in the vicinity of the colony (e.g. murres could forage for sympagic prey on the undersides of sea ice). As such, while ice use indices and to some extent HBI distributions are correlated to sea ice concentration around PLI, it seems that HBI abundances reflect the accessibility of prey to murres which, at least in this study, are associated to ice “type” and configuration. In 2012, the distribution of HBIs in murre eggs highlighted two distinct groups: one made by birds more associated with sea ice and one where eggs contained more of the phytoplankton-derived triene. Like earlier in 2002^42^, 2012 can be considered as an extreme year regarding ice conditions, with no access to open water except at the ice edge located more than 250 km away to the east. The only other access to open water was a small polynya located approximately 130 km southwest from PLI (Fig. 1). Our results thus suggest that some birds might have headed south to this small polynya while other birds might have headed east to feed in open waters out of Lancaster Sound and the north Baffin Bay. This reflects contrasting responses of birds to adverse foraging conditions. Such plasticity in bird behaviour was already reported by Pratte *et al*.^53^ who showed that, facing such extreme ice conditions, seabirds can adopt different strategies. Grémillet and co-workers^54^ also showed that the great plasticity of little auk (*Alle alle*) feeding behaviour was allowing them to maintain their fitness levels and attenuate effects of climate change.

While HBIs were quite abundant in murre eggs, the analysis of those laid by fulmars revealed much lower HBI abundances with, on average, concentrations approximately five times lower than those observed in murres (Supplementary Fig. S2). Cusset and collaborators (unpublished) recently analysed different tissues (muscle, liver, blood) from fulmars and murres and, although HBIs were a bit less abundant in fulmars, they did not observe such extreme differences. It may be possible that HBI transfer to eggs in fulmars is not as efficient as in murres, but several factors may be involved. A plausible explanation would lie in the different breeding phenology of each species, with fulmars laying their egg a few weeks earlier than murres. In fact, fulmars form their egg at a time when phytoplankton just start to bloom, meaning that only very low amounts of triene are available to grazers and their predators. Also, although sympagic algae are blooming, these are not released from the ice and therefore IP_25_ and diene are relatively unavailable to higher trophic levels. Indeed, high abundances of HBIs were reported in copepods collected in spring in Beaufort Sea^55^, but a three week lag between the sympagic bloom and HBI accumulation in zooplankton was observed. Further, even in the middle of winter (January), HBIs were observed in most compartments of the Ripfjorden ecosystem except those from above 75 m deep^56^. Since the highly mobile and opportunistic fulmars are restricted to surface waters, it is likely that only a small amount of HBIs are available during their pre-laying period. As such, the large differences in HBI abundances in murre and fulmar eggs most likely originate from their distinct and contrasting trophic ecologies. Fulmars are capable of travelling over large distances^57^ (>250 km) to reach their feeding grounds, and in our study region they can fly >500 km one way to feed^58^. Furthermore, during the pre-laying period, fulmars undertake their pre-laying exodus, during which they leave the colony and probably travel out of Lancaster Sound to the eastern Baffin Bay or potentially the North Water Polynya^58^, where open-water is more predictable across years and algal blooms may occur earlier. Thus, fulmars are easily able to reach open waters even during extreme years, and this could explain the lack of interannual differences in this species. In this case, studying GPS-equipped birds would help refining their actual feeding grounds and adjusted analyses of ice conditions in this particular area would help clarify the influence of sea ice for northern fulmars.

Thick-billed murres are pursuit-divers, diving down to 200 m depth to feed mainly on fish, especially the ice-associated Arctic cod (*Boreogadus saida*, Lepechin), and macrozooplankton^47^. Their feeding range is much shorter than the fulmar’s and typically restricted to approximately 150 km from their colony^59,60^. Murres are often observed near ice edges^61^ where they can easily dive under sea ice to forage on sympagic prey. Northern fulmars are opportunistic surface-feeders relying on fish such as arctic cod, but also on copepods, mysids, and gelatinous prey^62–66^. Given such differences in their feeding ecology, it is not surprising to see that H-Print and the ice use index differ between the two species. For thick-billed murres, both indices were closely linked to local ice concentrations and varied linearly with mid-June ice concentrations within a 100 km radius circle from the colony. For northern fulmars, neither H-Print nor ice use index were influenced by ice conditions. However, these interspecific differences might be explained by the fact that ice concentrations are defined in mid-May and mid-June for fulmars and murres, respectively. Ice concentrations exhibited much higher interannual variability during mid-June (29–99%) than during mid-May (80–99%). Hence, in our study fulmars did not experience years with reduced ice cover during egg formation. It is thus reasonable to expect smaller variations in HBI concentrations and distributions and, therefore, a weaker correlation to sea ice conditions for fulmars. Besides, fulmars have a longer breeding period, and Mallory and Forbes^67^ suggested that the high Arctic is near the environmental limit at which fulmars can complete their breeding. In contrast, thick-billed murres naturally lay eggs at a time closer to seasonal ice retreat and are hence already exposed to larger variations in ice conditions. Thus, the much lower interannual variations in sea ice cover might be below the threshold for northern fulmars to be influenced by sea ice variations (as they are constrained to breed early and fly far to complete chick-rearing before migration), and maybe that only extremely early open-water conditions (e.g. as early as in mid-May) would lead to observable impacts.

### Do seabirds change their feeding habits to cope with changes in ice conditions?

Variations in sea ice conditions clearly influenced the feeding ecology of both species. However, as for the usage of ice-derived resources, there appear to be large interspecific differences (Supplementary Fig. S3). As ice concentrations increased, eggs laid by murres exhibited a higher δ^13^C content and therefore probably reflected the fact that birds were relying more on sympagic resources. With increasing sea ice, murres also seemed to feed on prey from higher trophic levels (i.e. higher δ^15^N). The average increase of 0.7‰ in δ^15^N observed between 2011 and 2010 (i.e. years with the lowest and highest values on average, respectively), however, is too low to reflect a change in trophic level but rather suggests a higher percentage of prey of higher trophic level in their diet. Higher δ^15^N values are correlated to low H-Print. The presence of ice in the vicinity of the colony favors murre access to prey belonging to higher trophic levels and exhibiting a stronger relationship to sea-ice derived resources. Inversely, in years with reduced ice coverage, murres prey on organisms from lower trophic levels such as hyperiid amphipods (*Parathemisto spp*.), mysids or copepods^63,68^ that are more associated with pelagic resources. Our data confirm that murres take advantage of sea ice for feeding and thus any variation in ice cover has an influence on its accessibility to prey. Interestingly, δ^15^N values obtained from 2012 egg samples are lower than those of 2010, even if 2012 was the iciest year of the study. Again, as with HBI abundances, nitrogen isotopic values suggest that in 2012, ice conditions were too harsh to provide birds with easy access to their preferred fish resources. This suggests that an intermediate ice cover during egg formation represents the optimal breeding (foraging) scenario for murres at this colony. Indeed, arctic cod hide under the ice to avoid predators^69^, and during years like 2010 where ice was abundant but highly fragmented, birds could easily access their fish prey. In extreme years like 2012, ice precludes access to much of the sympagic prey for murres, and birds would forage more in the open water, spending much energy while focusing on fish, and had also to rely on zooplankton.

In contrast, high and relatively invariable δ^13^C values in eggs laid by fulmars indicate that birds fed in coastal icy habitat regardless of ice conditions. Lower (than murres) and invariable δ^15^N values also indicate that fulmars preyed upon lower trophic levels (likely zooplankton), without any influence of ice conditions. This absence of variation in isotopic composition has also been observed recently^53^ and suggests that every year, birds reach the same “predictable” feeding areas. These are probably located along the western Greenland coast in Baffin Bay, as suggested by tracking of foraging fulmars from a colony farther north on Devon Island^58^.

### Impact of sea ice on seabird egg parameters

In the current context of sea ice decline in the Arctic, studies focusing on the relationships between bird reproductive performances and ice are needed^70^. Indeed, top-predators such as birds closely synchronize their breeding schedule with their food supply in order to meet the energetic needs of their reproduction^71,72^. In the Arctic, food supply is closely linked to sea ice since both phytoplankton and sea ice algal blooms are controlled by its presence or absence^73^. In our study, we used egg size (i.e. egg volume) as a proxy for breeding investment. Indeed, a number of studies showed that although energetically costly to produce, larger eggs produce bigger chicks which then have a greater survival potential^74–76^. For murres and fulmars, Gaston and collaborators^42^ performed an exhaustive study and monitored colonies of both species at PLI between 2000 and 2003. They showed that in 2001 and 2002 (years relatively similar to 2012) laying was delayed and thick-billed murres produced smaller eggs. This was also reported by Hipfner and colleagues^49^ who showed that thick-billed murres laid smaller eggs later in the season during years of extensive sea ice in the High Arctic (PLI and Coburg Island). Both studies therefore showed that heavy ice conditions were leading to reduced breeding success, delayed laying date and decreased body condition/growth of chicks. In our study spanning very contrasting ice conditions, we observed an opposite effect of sea ice on murre breeding investment, with eggs on average larger in icy years than in years of open water. In previous studies, sea ice was only considered as a physical barrier that forces birds to commute over larger distances and involves higher energetic costs for breeding adults^42^. Hence, ice conditions were defined only according to the distance between colony and ice margin and did not consider the type of ice and how it was distributed (e.g. fast ice *versus* pack ice) as in the present study. Here, ice concentration around PLI, the position of the margins and the breakout period in 2010 and 2012 were relatively similar. However, as described above, the situation is extremely different when it comes to accessibility to food resources. In 2010, the very fragmented ice pattern provided murres with easy access to open water. In contrast, in 2012, birds had to travel over very long distances to reach open water. This situation prevented them from fully relying on ice-associated resources. The correlation between egg size and ice use index illustrate these differences in resource accessibility and use. Larger eggs were more associated with ice-derived resources (i.e. lower H-Print and higher ice use index) and therefore sea-ice conditions impacted the size of thick-billed murres eggs by modulating the availability of sympagic prey. Goutte *et al*.^77^ reported similar findings from their study on Adélie penguins (*Pygoscelis adelia*). They also highlighted a positive impact of a higher contribution of ice-derived resources on the breeding success of this species during years of extensive ice cover. Like murres, the Adelie penguin is an ice-associated diver that feeds mainly on Antarctic krill (*Euphausia superba*) and fish such as *Pleurogramma antarcticum*^78^, in waters covered by 20 to 80% ice as well as in the open sea, under pack ice or under coastal fast ice^79^. Guen *et al*.^80^ showed that intermediate ice coverage (20% ice), provided optimal breeding for Adelie penguins since krill juveniles rely exclusively on sympagic flora and the presence of sea ice promotes higher abundances of krill^81^. However, Emmerson and Southwell^82^ also showed that, as for murres, sea ice and, in particular, the presence of fast ice close to the shore could negatively impact reproduction performance of Adelie penguins by reducing their access to prey items. Thus, prey availability (accessibility and/or quality) clearly influences the breeding performance of various seabird species.

In subarctic areas, like in Hudson Bay, a decrease in the breeding success of murre colonies resulted from an increase in relative abundances of capelin (*Mallotus villosus*) in southern Arctic waters at the expense of Arctic cod^41^. A switch from Arctic cod to demersal sculpins, resulting from shifts from arctic to subarctic regimes, was also observed in black guillemots (*Cepphus grille mondtii*) in the Beaufort Sea and associated with a decrease in chick condition^83^. For seabird species laying a single egg, quality and quantity of food delivered by parents to the chick has a strong effect on the reproductive success^84^. In pigeon guillemots (*Cepphus columba*), chick growth and survival decreased as the proportion of the lipid-rich Pacific sand lance (*Ammodytes hexapterus*) decreased and as the proportion of low-lipid demersal fish increased^85^. Here, we provide evidence that ice-derived resources influence murre egg size (proxy for breeding investment) and further investigations including other breeding parameters (such as chick growth rate and survival, breeding success) would help to confirm the observed trends and understand murre vulnerability to sea ice declines in the Arctic.

The energy content of murre eggs was greater with higher use of ice resources and when the birds fed at a higher trophic level, suggesting higher investment in eggs during icier years. Furthermore, larger eggs contained more energy for the chick’s development, ultimately providing nestlings with higher chances of survival. Barrionuevo and Frere^74^ showed that yolk-area was a strong predictor of nestling survival in another diving seabird, the Magellanic penguin (*Sheniscus magellanicus*), with yolk-area positively related to nestling survival, and nestling body size positively impacted by egg volume. Larger eggs with larger yolk contain more lipids and more energy for the chick’s development, producing ultimately a larger chick with higher chances of survival^86^. Therefore, sea ice represents a real and valuable asset for ice-associated seabirds, like murres, that might be more sensitive to future changes in Arctic sea ice.

For northern fulmars, egg size remained stable (Fig. 5), without any impact of either sea ice concentrations or ice-derived resources, consistent with previous results (same laying date, same egg size)^42^. Unlike murres, fulmars thus appear less sensitive to sea ice variations which do not seem to impact their investment in reproduction. As such, northern fulmars are more likely to be dramatically affected by a « tipping point » in Arctic sea ice conditions at the time of egg development in females; a point at which previously small changes become significant enough to impact their feeding ecology and reproductive investment. Further investigations on fulmars during years of highly variable ice conditions are thus required to fully understand the influence of sea ice for this species.

## Conclusion

Our study examined the importance of sea ice and ice-derived resources for breeding Arctic seabirds in a context where the Arctic might become ice-free in summer within the next decades. Instead of considering the sea ice cover as a physical barrier preventing seabirds to access their prey, we highlight the importance of sea ice *via* the resources it provides to marine predators. Our multi-biomarker approach, combining highly branched isoprenoids and stable isotopes, showed how important sea ice is for seabirds and how its use conditions different aspects of their biology, including their egg characteristics. For the fulmar, which has exceptional ability to fly long distances but must breed early to complete its long breeding period, variation in sea ice had limited effects, presumably because birds have adapted to fly long distances to open water remote from the colony but predictably open early in the season. However, the influence of sea ice is especially important for species such as diving thick-billed murres, perhaps more constrained in their response to sea ice variations, that rely heavily on local environmental conditions. At least for this colony, murres appear to have “optimal” ice conditions when ice cover is heavier but still broken up providing feeding locations; ice-associated biomarkers and proxies of reproductive effort are reduced at higher or lower ice conditions. Overall, we provide new and essential knowledge to comprehend the consequences of current and future climate changes on the fate of Arctic seabird populations. We also emphasise the importance of combining different biomarkers to better understand the importance of sympagic resources for top predators within changing Arctic marine ecosystems.

## Methods

### Sample collection

As part of a long-term monitoring program supported by Environment and Climate Change Canada and the Northern Contaminants Program, eggs of murres and fulmars were collected during four consecutive years (2010–2013) from nests on the eastern and southern cliffs at the multi-species colony on Prince Leopold Island in Lancaster Sound (74°N, 90°W; Nunavut). For consistency, murre and fulmar eggs were sampled at the same period every year (late June – early July). One egg per nest was randomly sampled shortly after laying for murres and at mid-incubation for fulmars (murres and fulmars generally lay only one egg). Eggs were either taken by hand or by using an extension pole holding a small cup. In the field, eggs were kept cool and shipped to the National Wildlife Research Center (Ottawa) for processing. All eggs were taken under appropriate annual research and collection permits (e.g. Nunavut Wildlife Research Licence 2012–040; Environment Canada NUN-SCI-12-04). Egg length (mm), width (mm) and weight (g) were measured, and a volume index (cm³) was calculated according to previously published methodology^42,47^ (Eq. 1).

1 $${\rm{Volume}}={\rm{Length}}\times {{\rm{Width}}}^{{\rm{2}}}\times {10}^{-3}$$

Egg contents were homogenized and stored frozen at −40 °C in acid-rinsed polyethylene vials. One- or two-gram aliquots (15 eggs per species per year) were sent to Laval University (Quebec City) where HBI and caloric content analyses were performed.

### Environmental conditions

As murres have a local feeding range (0–150 km^59^), we described environmental conditions by characterizing the presence/absence of sea ice, its type (size, thickness, presence of leads) and concentration in a 100 km radius circle around Prince Leopold Island. To determine the presence and the type of sea ice around the colony, regional ice maps (i.e. Eastern Arctic; weekly total concentrations; week including 20 June) were downloaded from Canadian Ice Service Archives for the 2010–2013 period. Ice maps for the 2000–2003 period were also downloaded in order to compare our data with a previous study^42^. As these maps gather weekly data at a regional scale, we used satellite images from NASA Worldview dataset (EOSDIS, https://worldview.earthdata.nasa.gov/) to determine sea ice distribution and concentration at a finer temporal and spatial scale. The proportion of sea ice around the colony was calculated using MODIS 500 m resolution raster images and only images with clear sky were considered. Pixels were first extracted in a circle of 100 km radius around the colony. Land pixels were then masked out using Open Street Map mask (http://openstreetmapdata.com/data/water-polygons). The remaining pixels were classified into two categories (open water or sea ice) using a K-means clustering procedure. The clustering has been performed using the RGB values of the pixels. A maximum of 100 iterations was allowed for convergence. The proportion between pixels classified as open water and sea ice was then calculated and used as a proxy for the proportion of sea ice around the colony.

### HBI analyses

HBIs were analysed following the procedure initially described in Belt *et al*.^87^. Samples were freeze-dried for 48 h and the water content determined. 7-hexylnonadecane and 9-octylheptadec-8-ene (10 µL; 9,68 µg.mL^−1^ each) were added as internal standards to dried aliquots (~0.1 g to 0.3 g dry weight). Total lipids were extracted four consecutive times with dichloromethane-methanol mixture (2:1, 15 min sonication). Delipidized samples were dried overnight at 45 °C (12 h) and stored frozen (−20 °C) for isotopic analyses. Total lipid extracts were then dried under a gentle stream of N_2_ and subjected to saponification (4 mL, 5%, MeOH/H2O, 90/10, 90 °C, 1 h). Non-Saponifiable Lipids (NSL) were extracted from saponification mixture by liquid-liquid extraction (Hexane, 3 × 2 mL), evaporated (nitrogen stream <35 °C) and purified using open column chromatography (SiO_2_ 50 g.g^−1^ NSL; Hexane) to yield an apolar lipid fraction containing HBIs. These apolar fractions were then analysed *via* GC-MS following the procedure described in Belt *et al*.^87^. Briefly, the abundance of IP_25_, diene and triene were determined to integrate 350.3, 348.3 and 346.3 responses, respectively. The ratio of these responses against those of internal standards (m/z 266) was normalized to the mass of the sample and multiplied by the mass of standard added to the sample. For IP_25_, response factors were determined by injection of authenticated standards and results are expressed in ng.g^−1^. For other HBI isomers, in the absence of pure standards results are expressed in ng of standard equivalent.g^−1^.

Once individual HBIs were identified and quantified, the H-Print index was calculated for each sample using Eq. (2) from Brown *et al*.^31^:

2 $${\rm{H}}-{\rm{P}}{\rm{r}}{\rm{i}}{\rm{n}}{\rm{t}}({\rm{ \% }})=[{\rm{t}}{\rm{r}}{\rm{i}}{\rm{e}}{\rm{n}}{\rm{e}}/({{\rm{I}}{\rm{P}}}_{25}+{\rm{d}}{\rm{i}}{\rm{e}}{\rm{n}}{\rm{e}}+{\rm{t}}{\rm{r}}{\rm{i}}{\rm{e}}{\rm{n}}{\rm{e}})]\times 100$$

where 0, 50 and 100% correspond respectively to a sympagic (i.e. ice algae), a mixed (i.e. ice algae and phytoplankton) and a pelagic (i.e. phytoplankton) association.

### Stable isotope analyses

Stable isotope analyses were performed on aliquots of the delipidized fractions resulting from HBI analyses and loaded into tin cups (0.2 to 0.8 mg dry weight). An elemental analyser (Flash EA 1112, Thermo Fisher) coupled in continuous flow mode to an isotope ratio mass spectrometer (Delta V Advantage, Thermo Fisher, Bremen, Germany) was used to determine stable isotope abundances. Results were expressed in δ notation as the deviation from standards in parts per thousand (‰), according to Eq. (3):

3 $${\rm{\delta }}{\rm{X}}=[({\rm{R}}({\rm{sample}})/{\rm{R}}({\rm{standard}}))\mbox{--}1]\times {10}^{3}$$

where X is ^13^C or ^15^N, and R is the corresponding ratio ^13^C/¹²C or ^15^N/^14^N. Standard values were obtained from Vienna Pee Dee Belemnite (VPDB) and atmospheric N_2_ (air) for C and N respectively. Stable isotopes analyses were performed at the Littoral, Environment and Societies institute (LIENSs, France). Replicate measurements of laboratory standards (USGS-61 and USGS-62) indicated that the measurement accuracy was <0.2% for both δ^15^N and δ^13^C values (analytical precision <0.15%). δ^15^N and δ^13^C isotopic values provide information on the relative trophic level of birds (i.e. diet) and their feeding habitat (i.e. carbon source) respectively^88,89^. Based on distinct δ^13^C signatures of ice algae and phytoplankton (i.e. more enriched ice algae), δ^13^C is also considered as an ice proxy in the Arctic^26,27^.

### Egg caloric content analyses

Aliquots of dried homogenized samples (*n* = 32) were compressed to form pellets and analysed in a Parr 6300 Automatic Isoperibol-Oxygen Bomb Calorimeter. A Benzoic Acid standard was added to each sample (0.3–0.7 g) due to low sample weight (<0.2 g). Instrument precision was determined by running Benzoic Acid standards (6318 cal.g^−1^) prior to the samples. Results were expressed in both cal.g^−1^ dry and wet sample. The energy available per egg (kcal) was calculated by multiplying cal.g^−1^(wet) with the total egg weight. Unfortunately, the weight of the shell of each egg was not determined prior to homogenization and we, therefore, make the assumption that the variation amongst individuals is negligible.

### Statistical analysis

A principal component analysis (PCA) was performed using the two sea-ice tracers (H-Print and δ^13^C) in order create a single variable representing bird-ice association (hereafter named Ice Use Index). The first principal component (PC1) explained 78.4% and 79.3% of the total variance in murres and fulmars, respectively, representing a range of sea-ice use, on which individual coordinates were projected. To investigate the influence of ice association on bird feeding ecology and reproductive parameters, we tested linear or quadratic regressions of δ^15^N, egg volume and energetic content with the Ice Use index. Normality of residuals and homoscedasticity were tested prior to interpretation using the Shapiro-Wilk normality test and a Breusch-Pagan test, respectively, and data were log-transformed when assumptions were not respected.

For each species, a Multivariate Analysis of Variance (MANOVA) was performed to test whether there were significant differences in the isotopic niche (δ^13^C, δ^15^N) of birds between years. One-way ANOVA then enabled to test significant differences of each variable between years, and posthoc Tukey HSD tests were performed to locate these differences. Residuals normality and variance homogeneity were tested prior analysis with tests described above, and data were log-transformed when these assumptions were not respected.

Due to small sample sizes, we used Welch-modified *t*-test to distinguish groups of murres in 2012 (H-Print and egg volume), for interspecific differences (δ^15^N) and for inter-annual differences in egg volume and energetic content between reduced-ice (i.e. 2011 and 2013) and extensive-ice years (i.e. 2010 and 2012). All statistical analyses and figures were computed in R^90^ (Version 3.5.0).

### Ethical approval

All applicable institutional and national guidelines for the care and use of animals were followed and methods were carried out in accordance with relevant guidelines and regulations, including appropriate scientific and land use permits. All eggs were taken under appropriate annual research and collection permits (e.g. Nunavut Wildlife Research Licence 2012–040; Environment Canada NUN-SCI-12-04). The local indigenous community was consulted and had representatives participate in the work in most years.

## Supplementary information

Supplementary Information

## Data Availability

The datasets generated and analysed during the current study are available from the corresponding author on a reasonable request.
