# Supplementary material for: Arctic seabirds and shrinking sea ice: egg analyses reveal the importance of ice-derived resources
Source: Sci Rep. 2019 Oct 28;9:15405. doi: 10.1038/s41598-019-51788-4 (PMC6817817; doi:10.1038/s41598-019-51788-4)
Supplement: Supplementary file 1 — Supplementary Information [file 41598_2019_51788_MOESM1_ESM.pdf]

Arctic seabirds and shrinking sea ice: egg analyses reveal the importance of ice-derived resources

Fanny Cusset, Jérôme Fort, Mark Mallory, Birgit Braune, Philippe Massicotte and Guillaume Massé

Supplementary figures

S1. (a) Satellite images of Barrow Strait (Nunavut, Canada) showing ice conditions around Prince Leopold Island (inner red circle) during the breeding season of northern fulmars (NOFU) and thick-billed murres (TBMU) (from EOSIDS Worldview dataset). The outer red circle represents the 100 km radius circle around the colony. Each row represents a key reproductive event for both fulmars and murres, from their arrival at the colony (in early-May and mid-June respectively) to egg-laying (in early-June and early-July respectively) and egg hatching (in end-July for both species). Only fulmars undertake a pre-laying exodus: they leave the colony temporarily between colony attendance and egg-laying. 2010 and 2012 appear as icy years, inversely to 2011 and 2013 during which open water was dominant. **(b)** Daily ice concentrations in the 100 km radius circle around Prince Leopold Island between May and September for the 2010-2013 period. Red dots correspond to dates presented above.

S2. HBI distributions (ng g^{-1} standard) in eggs of thick-billed murres (a) and northern fulmars (b) (*n*=15 per year and species) collected on Prince Leopold Island for four consecutive years with contrasted ice conditions (2010-2013).

S3. HBI concentrations (Mean ± SE, expressed in ng g^{-1} standard) in eggs of thick-billed murres (top) and northern fulmars (bottom) between 2010 and 2013. IP_{25} and diene (dark and light grey respectively) are ice biomarkers and triene (black) is a pelagic biomarker.

S4. Stable isotope results (Mean ± SD) for eggs of thick-billed murres (TBMU; black circles) and northern fulmars (NOFU; open circles) collected on Prince Leopold Island between 2010 and 2013. Numbers indicate the sampling size for each year and species.

S5. Influence of prey consumed by thick-billed murres ($\delta^{15}N$) on their egg energetic content (kcal).

S6. Relationship between volume (cm^3) and energetic content (kcal) in thick-billed murre eggs.

(a)

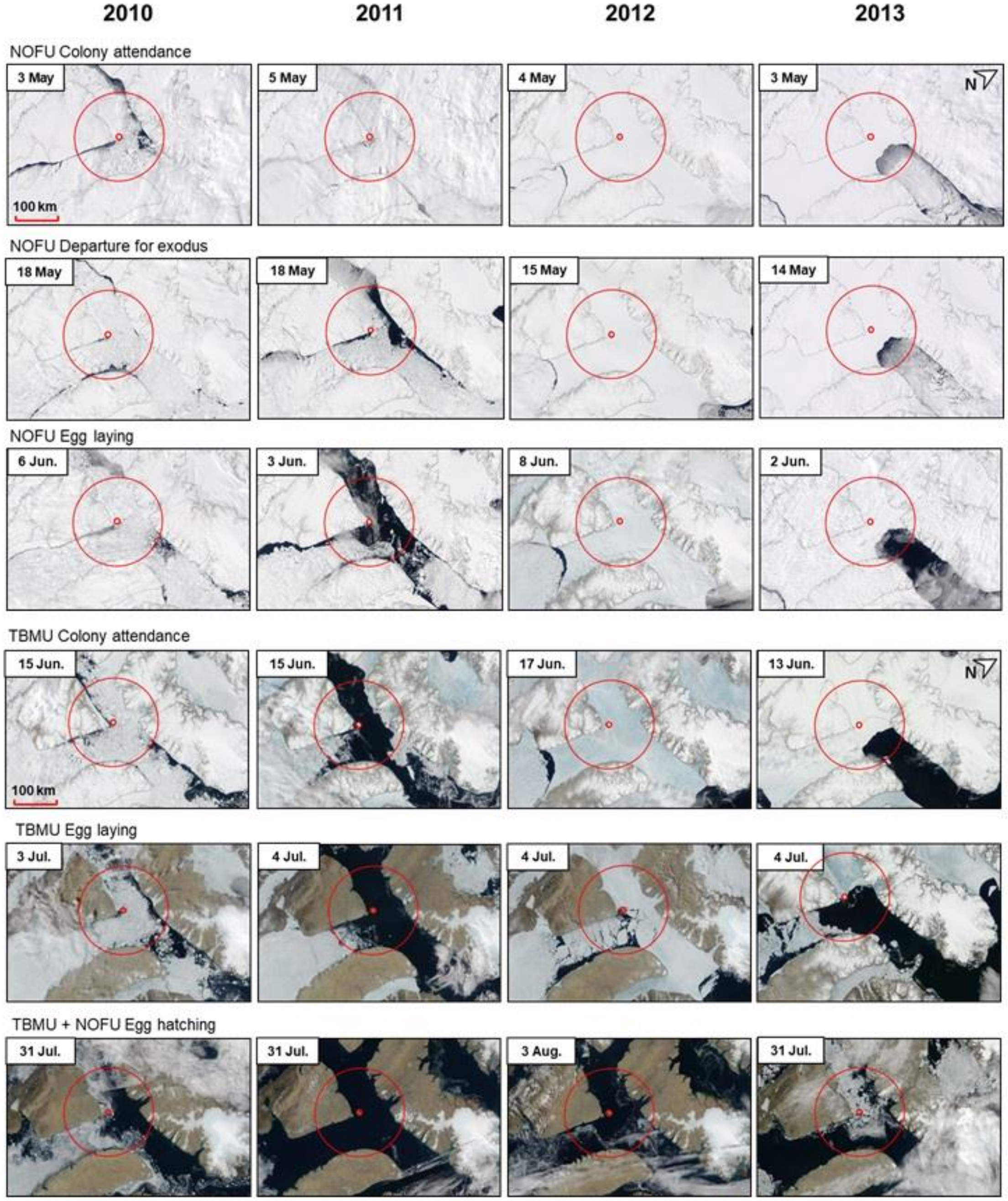
2010
2011
2012
2013
NOFU Colony attendance
3 May
5 May
4 May
3 May
N
100 km
NOFU Departure for exodus
18 May
18 May
15 May
14 May
NOFU Egg laying
6 Jun.
3 Jun.
8 Jun.
2 Jun.
TBMU Colony attendance
15 Jun.
15 Jun.
17 Jun.
13 Jun.
N
100 km
TBMU Egg laying
3 Jul.
4 Jul.
4 Jul.
4 Jul.
TBMU + NOFU Egg hatching
31 Jul.
31 Jul.
3 Aug.
31 Jul.

(b)

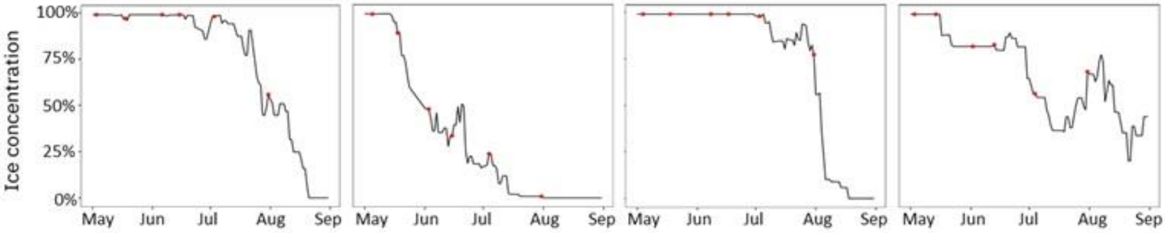
Ice concentration
100%
75%
50%
25%
0%
May
Jun
Jul
Aug
Sep

S1. (a) Satellite images of Barrow Strait (Nunavut, Canada) showing ice conditions around Prince Leopold Island (inner red circle) during the breeding season of northern fulmars (NOFU) and thick-billed murres (TBMU) (from EOSIDS Worldview dataset). The outer red circle represents the 100 km radius circle around the colony. Each row represents a key reproductive event for both fulmars and murres, from their arrival at the colony (in early-May and mid-June respectively) to egg-laying (in early-June and early-July respectively) and egg hatching (in end-July for both species). Only fulmars undertake a pre-laying exodus: they leave the colony temporarily between colony attendance and egg-laying. 2010 and 2012 appear as icy years, inversely to 2011 and 2013 during which open water was dominant. **(b)** Daily ice concentrations in the 100 km radius circle around Prince Leopold Island between May and September for the 2010-2013 period. Red dots correspond to dates presented above.

a) Thick-billed murres

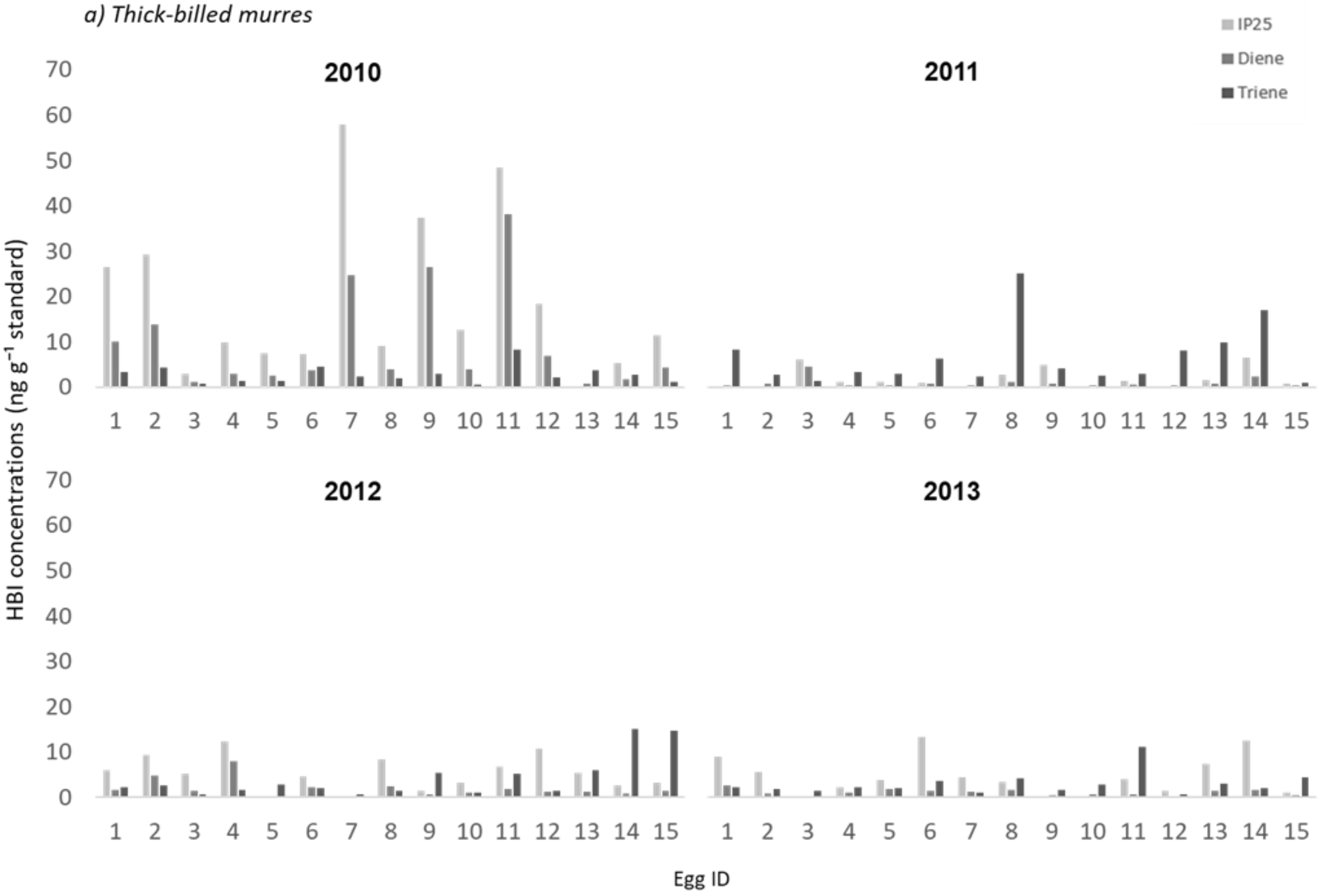

b) Northern fulmars

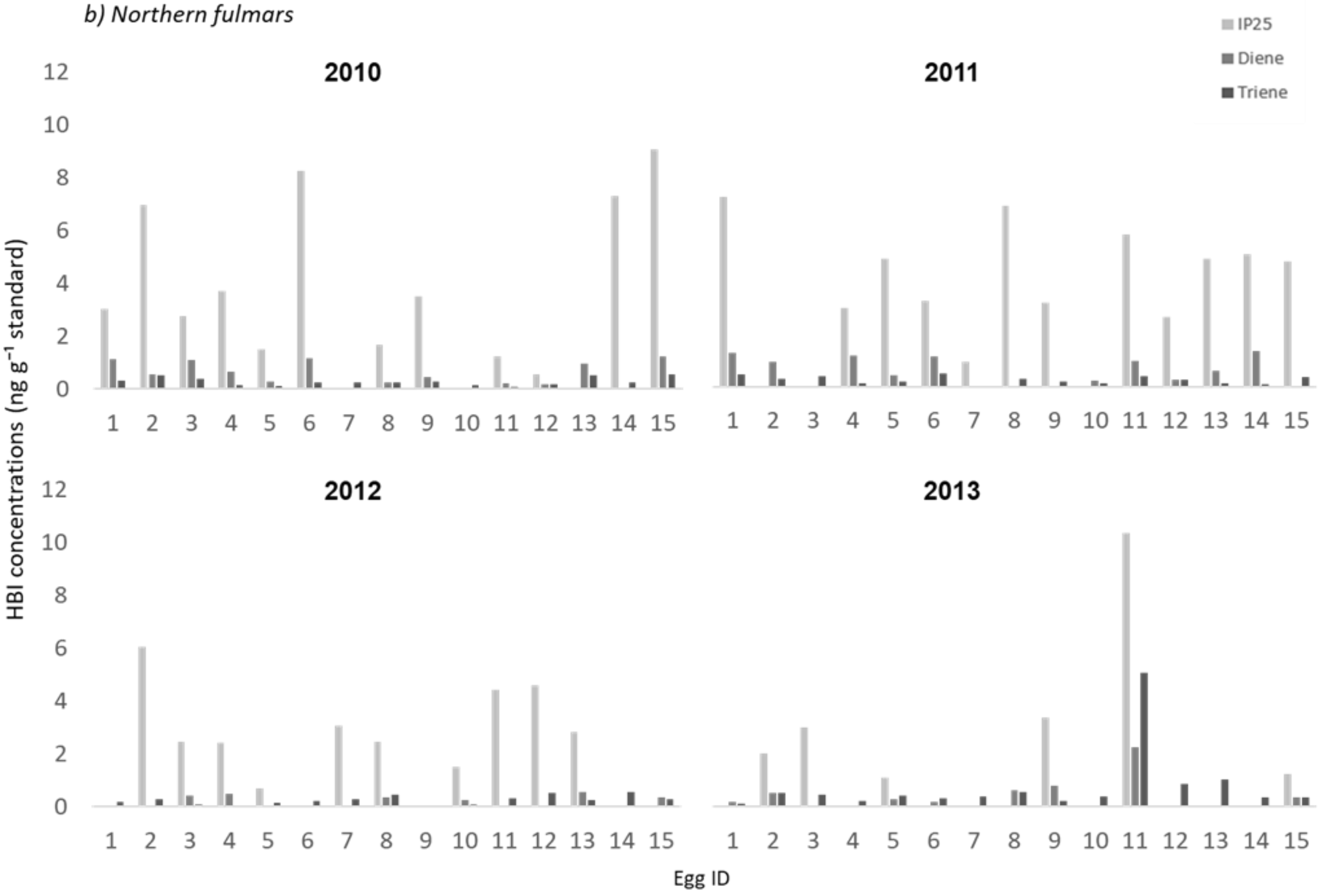

S2. HBI distributions (ng g⁻¹ standard) in eggs of thick-billed murres (top panels) and northern fulmars (bottom panels) (n=15 per year and species) collected on Prince Leopold Island for four consecutive years with contrasted ice conditions (2010-2013).

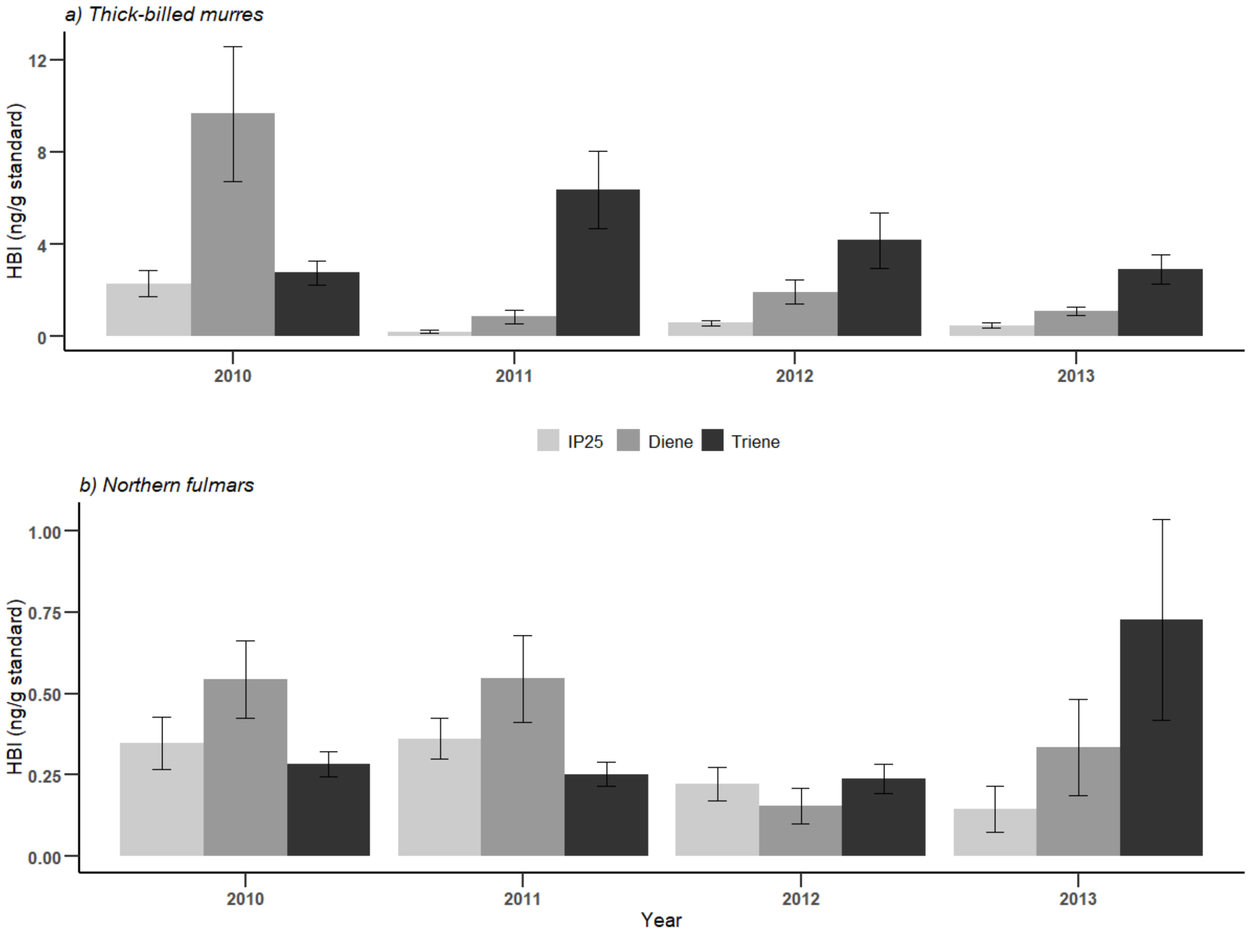

S3. HBI concentrations (Mean ± SE, expressed in ng g^{-1} standard) in eggs of thick-billed murres (a) and northern fulmars (b) between 2010 and 2013. IP_{25} and diene (dark and light grey respectively) are ice biomarkers and triene (black) is a pelagic biomarker.

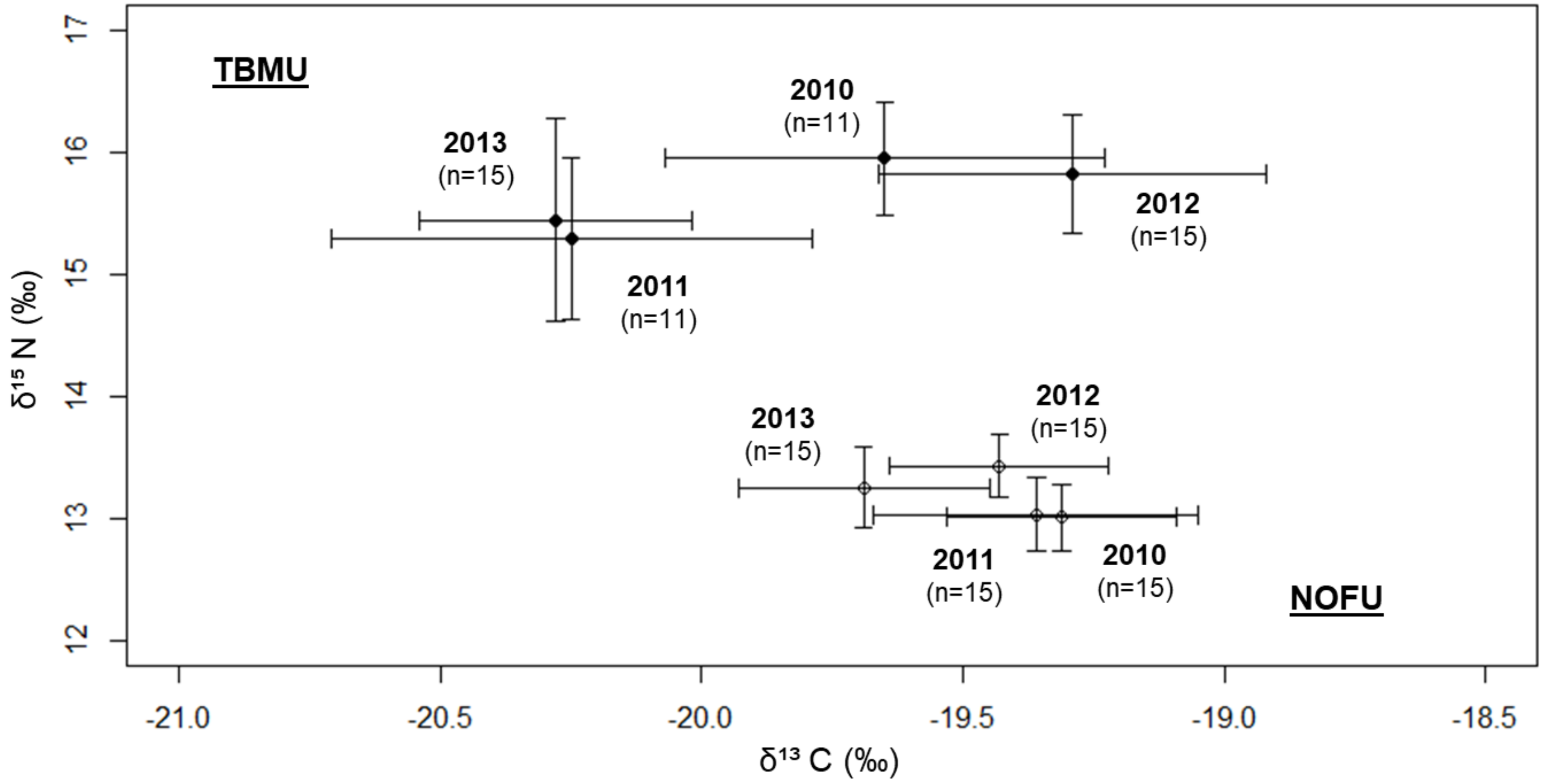

S4. Stable isotope results (Mean ± SD) for eggs of thick-billed murres (TBMU; black circles) and northern fulmars (NOFU; open circles) collected on Prince Leopold Island between 2010 and 2013. Numbers indicate the sampling size for each year and species.

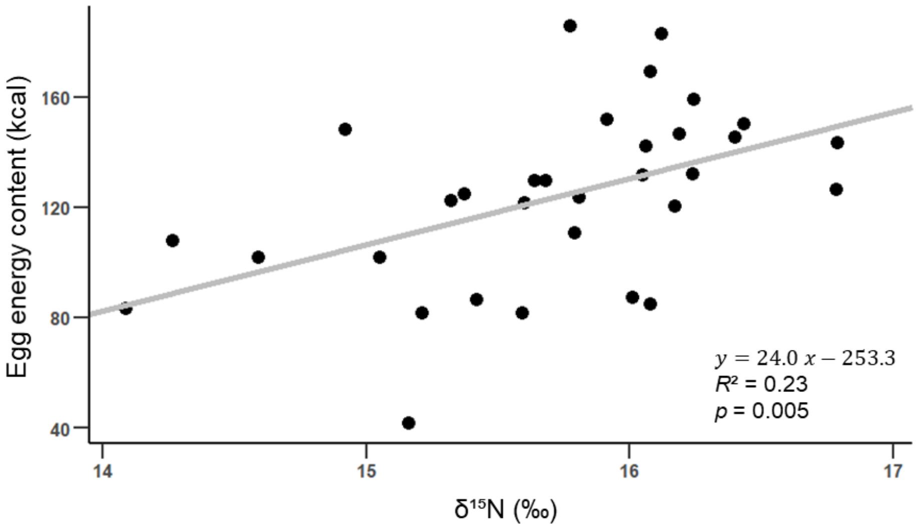

S5. Influence of prey consumed by thick-billed murres ($\delta^{15}N$) on their egg energetic content (kcal).

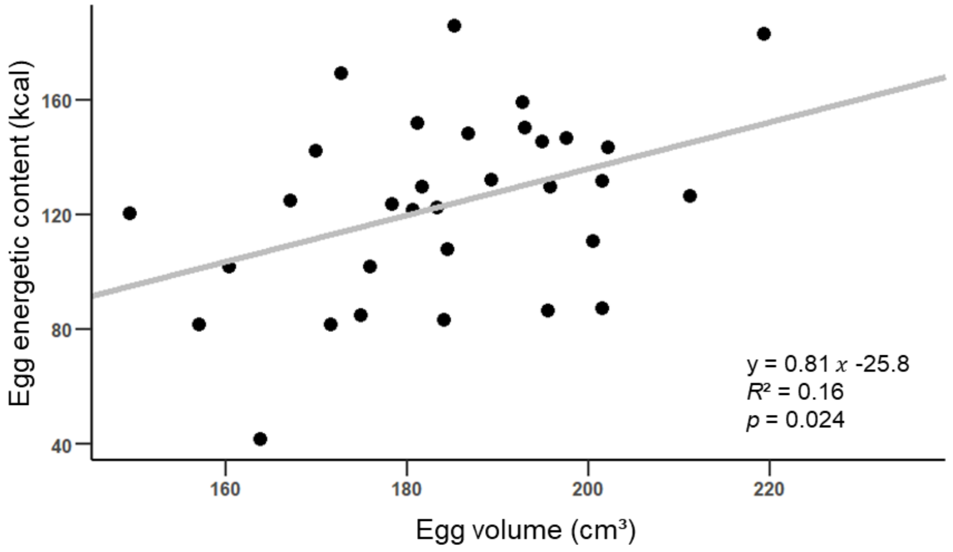

S6. Relationship between volume (cm^3) and energetic content (kcal) in thick-billed murre eggs.
